# Rituximab for Remission Induction and Maintenance in Refractory Systemic Lupus Erythematosus

**DOI:** 10.1155/2014/731806

**Published:** 2014-01-16

**Authors:** Fabio Bonilla-Abadía, Nicolás Coronel Restrepo, Gabriel J. Tobón, Andrés F. Echeverri, Evelyn Muñoz-Buitrón, Andres Mauricio Castro, Mercedes Andrade Bejarano, Carlos A. Cañas

**Affiliations:** ^1^Rheumatology Unit, Fundación Valle del Lili, ICESI University, Carrera 98 18-49, Cali, Colombia; ^2^Internal Medicine Unit, Fundación Valle del Lili, CES University, Cali, Colombia; ^3^Clinical Research Unit, Fundación Valle del Lili, Cali, Colombia; ^4^School of Statistics, Universidad del Valle, Cali, Colombia

## Abstract

Systemic lupus erythematosus (SLE) is a chronic inflammatory autoimmune disease with high morbidity if untreated. Sometimes, despite aggressive treatments, the disease remains active with cumulative organic damage. We conducted a retrospective and descriptive observational study of patients with SLE refractory to conventional treatment who were treated with rituximab (RTX) as remission induction therapy and maintenance. There was a significant reduction in the conventional immunosuppressive drug dose and the number of relapses of disease. RTX appeared to be effective and safe for the induction and maintenance of remission in patient with SLE refractory to conventional treatment.

## 1. Introduction

Systemic lupus erythematosus (SLE) is a chronic inflammatory autoimmune disease with no permanent cure or high morbidity if left untreated [1]. Sometimes, despite aggressive treatments with high dose of glucocorticoids and immunosuppressive drugs, the disease remains active with cumulative organic damage [2]. The disease severity appears to be higher in Hispanics compared to Caucasians. Thus, the overall survival rates vary by race and ethnic background with a 5-year survival rate of approximately 95% among whites, 90% among blacks, and 87% among Hispanics [3]. The management of patients with SLE depends on the type of organ involvement and severity of the disease. When manifestations are severe, with threatening life conditions, the treatment is based on high-dose steroids, plasmapheresis, intravenous gamma globulin, and various types of immunosuppressants such as cyclophosphamide, azathioprine, or mofetil mycophenolate [4]. Some patients remain with active disease despite full immunosuppressive drugs. Two monoclonal antibodies targeting B cells have been used successfully in refractory SLE: belimumab directed against B-cell activating factor (BAFF) [5] and Rituximab which is a genetically engineered chimeric anti-CD20 monoclonal antibody. CD20 is a B-cell surface antigen that is expressed only on pre-B and mature B cells. It is not present on stem cells and is lost before differentiation of B cells into plasma cells. Therefore, rituximab causes a selective transient depletion of the CD20+ B-cell subpopulation [6]. Rituximab (RTX) is currently approved for the management of B-cell lymphomas, rheumatoid arthritis (RA) and systemic vasculitis ANCA (antineutrophil cytoplasmic antibodies) positive [7, 8], and it is used as a single dose for SLE crisis with varying results [9–11]. There are few reports in the literature about routine and chronic use of RTX as a maintenance drug therapy in SLE [12]. Being a chronic and incurable disease with high rates of relapse, we decide to treat indefinitely a group of patients that previously had favorable response to single doses. In this study, we evaluate the efficacy and safety of RTX both as a rescue and maintenance agent in a group of patients with refractory SLE.

## 2. Patients and Methods

The data was taken from the electronic medical records of patients at a fourth-level center (Fundación Valle del Lili) in Cali, Colombia, throughout twelve years (from August/2001 to April/2013). Patients were eligible for the study if they had provided authorization for review of their medical records, were older than 18 years old at the time of the study, had a diagnosis of SLE based on the criteria of the American College of Rheumatology [13], and had refractory SLE to conventional treatments: hydroxychloroquine, high dose steroids, cyclophosphamide, azathioprine, and mofetil mycophenolate, which despite receiving recommended doses and validated protocols in the adequate time persisted with active disease (defined as a SLEDAI score higher than 4) [14]. Patients with active infections or cancer were excluded from the study. Medical records were reviewed and demographic and clinical data, including the number of RTX cycles, frequency and severity of relapses, glucocorticoids doses, and type and doses of conventional immune-suppressor agents, were collected. Assess of lupus activity was done with SLEDAI score [15]. Patients with refractory SLE were treated with RTX both as rescue medication for lupus flare and for maintenance (dose of 1 gr at day 0 and 1 gr at day 15 with retreatment every 9 months). Administration of acetaminophen 1 g, diphenhydramine 50 mg, and prednisone 50 mg prior to each RTX infusion was done. RTX was given as additional agent to the treatment received. We did not use the CD20+ cell counts as an indicator of the moment to retreat these patients.

### 2.1. Statistical Analysis

An exploratory analysis of the data was made using percentages for categorical variables and medians (interquartile range (IQR)) for continuous variables. The Wilcoxon nonparametric test was done for comparisons. Data analysis was done using STATA 1.2. software. *P* values less than 0.05 were considered significant for all statistical tests. The study was approved by the ethics committee of Fundación Valle del Lili research center.

## 3. Results

**Figure 1:**
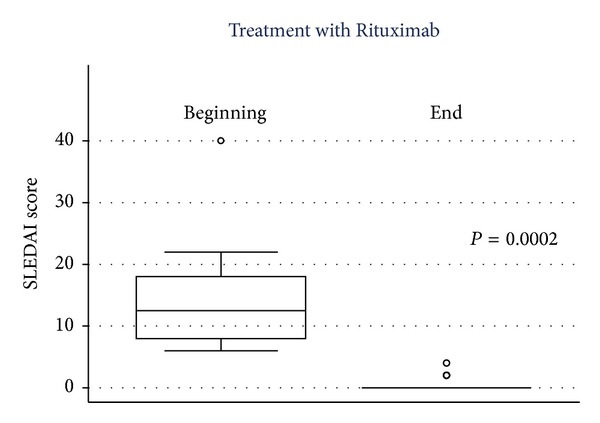
Box plot diagram of the SLEDAI score at the beginning and at the end of the last cycle of RTX.

Out of 350 patients of the initial cohort, eighteen patients with refractory and active SLE were included in the analysis. Seventeen out of eighteen patients were women and all patients were Hispanic. The median age was 28.5 years (range: 22–36). At the time of inclusion, median SLEDAI score was 12.5 (range: 8–18). Median initial doses were as follow: prednisone, 25 mg/day (range: 15–20); mofetil mycophenolate, 2.0 gr/day; azathioprine, 100 mg/day (2 mg/kg/day); hydroxychloroquine, 200 mgr/day; and endovenous cyclophosphamide, 750 mgr each month. The mean follow-up was 37.5 months (range: 18–63) with a mean average of 5 RTX cycles (range: 3–8). Baseline clinical characteristics, treatment history, and refractory organ system involvement in these 18 patients at the time of their first RTX course are described in Table 1. At the end of follow-up, SLEDAI score was 0 in fourteen patients, 2 in three patients, and 4 in one patient (*P* = 0.0002)—(Table 2 and Figure 1). Median prednisone dose at the end of follow-up was 3.75 mg/day (range: 2.5–5) (*P* = 0.0002). In addition, mofetil mycophenolate and azathioprine were both discontinued in all patients (*P* = 0.0071 and *P* = 0.052, resp., compared to baseline), and hydroxychloroquine median dose decreased to 75 mgr/day. The median relapses rate at the beginning of RTX treatment was 3 per year (IQR 3–5), decreasing to 0 (IQR 0-1) at the end of follow-up.

Because the use of RTX in rescue dose in our patients with acute disease was favorable in all but one, we decided to retreat them every nine months.

Three patients presented an adverse reaction to RTX at the first course of treatment consistent with cytokine release syndrome [16]. However, the application of the drug was not suspended in these cases, and a desensitization protocol was successfully performed in the hospital.

## 4. Discussion

Here, we present a case series that evaluated and demonstrated safety and efficacy of RTX therapy in induction and maintenance for the treatment of SLE.

Two randomized clinical trials have been published trying to prove the clinical effectiveness of RTX in SLE with mixed results. The EXPLORER study [9], a case-control study conducted in 257 patients with extrarenal SLE, showed no statistical significance in reduction in disease activity between RTX and conventional immunosuppressive therapy. The LUNAR study [10] randomized 144 patients to receive either RTX or placebo, under a mofetil-mycophenolate-based immunosuppression and steroids, showing a significant improvement in the levels of C3, C4, and anti-DNA but no differences in renal response rates at week 52 of treatment. Pinto et al. [11] conducted a prospective study of a cohort of 42 patients with refractory SLE in Colombia, adding RTX as a rescue therapy in one initial dose, with 36% of the patients showing complete remission and an overall significant reduction in steroid use. The preferred scheme used for ablation of B cells with RTX was an initial dose of 1 g and then 1 g in two weeks. Subsequent doses were not indicated. The excellent clinical response in Colombian patients may be explained by racial grounds, which has been shown in the present study and in other recent publications included Latin American population [17–19]. All patients including in our series were mestizos. More studies are needed to confirm these findings.

Our scheme was done based on the protocols used in RA patients and as now it is beginning to be recommended in refractory granulomatosis with polyangiitis [20]. All patients showed to the end of the study remission criteria. This follow-up study showed that depletion of B lymphocytes with repeated RTX is effective and safe in patients with SLE. A decrease in the number of relapses by disease activity was also evident. Relapses were prevented with RTX retreatment and conventional immunosuppressive doses were decreased gradually. Adverse events related to the infusion were few and there was no contraindication for retreatment with RTX. All patients in this cohort reached remission of the disease.

One of the most interesting findings and strengths of this study is that only few reports have shown the effectiveness of RTX retreatment in SLE patients, as used in a routine way in rheumatoid arthritis patients and not only at the moment of refractory involvement. Several shortcomings are presented in our study. First of all, this is a retrospective series, and no randomized assessment was done. In addition, only 18 patients were evaluated and no B-cell count was done to define the adequate moment to retreatment.

In conclusion, RTX seems to be effective and safe for the induction and maintenance of remission in Colombian mestizo patients with SLE refractory to conventional immunosuppressive therapy. Our results provide important information for the design of future studies in order to confirm the results obtained here.

## Figures and Tables

**Table 1:** Baseline characteristics of the 18 systemic lupus erythematosus patients and their main refractory and active involvement.

Characteristics	Values (*n* = 18)
Age, years*	28.5 (22–36)
Female, Gender	17 (94.4)
Prednisone dose*	25 (15–50)
Use of Azathioprine	5 (27)
Use of Cyclophosphamide	6 (33)
Use of Mycophenolate	9 (50)
Use of Hydroxychloroquine	11 (61)
Relapses/year*	3 (3–5)
Renal criteria**	11 (61.1)
Hematological criteria***	13 (72.2)
Cardiopulmonary criteria	8 (44.4)
Musculoskeletal criteria	14 (77.8)
Low C3	13 (72.2)
Low C4	10 (55.6)
Anti-DNA positive	9 (50)

All the data correspond to *n* (%) with the exemption of those marked with *.

*Median IQR (interquartile range).

**Proteinuria (higher than 0.5 g/day), casts (erythrocytes, hemoglobin, granular, tubular, or mixed), hematuria, and pyuria.

***Leukopenia (less than 4.000 cells/mm^3^), thrombocytopenia (less than 100.000 cells/mm^3^), and hemolytic anemia.

**Table:** (a)

Patient	SLEDAI score	
	Beginning	End
1	40	0
2	8	0
3	14	0
4	18	2
5	14	0
6	8	2
7	18	0
8	8	0
9	7	0
10	11	0
11	8	0
12	14	0
13	22	2
14	14	0
15	6	0
16	22	4
17	11	0
18	9	0

**Table:** (b)

	Beginning	End	*P* value
Score	12.5 (8–18)	0 (0-0)	*P* = 0.0002
